# NFκB signaling in alveolar rhabdomyosarcoma

**DOI:** 10.1242/dmm.030882

**Published:** 2017-09-01

**Authors:** Megan M. Cleary, Atiya Mansoor, Teagan Settelmeyer, Yuichi Ijiri, Katherine J. Ladner, Matthew N. Svalina, Brian P. Rubin, Denis C. Guttridge, Charles Keller

**Affiliations:** 1 Children's Cancer Therapy Development Institute, Beaverton, OR 97005, USA; 2 Department of Pediatrics, Oregon Health and Science University, Portland, OR 97239, USA; 3 Department of Pathology, Oregon Health and Science University, Portland, OR 97239, USA; 4 Department of Cancer Biology and Genetics and The Arthur G. James Comprehensive Cancer Center, The Ohio State University College of Medicine, Columbus, OH 43210, USA; 5 Department of Anatomic Pathology, Department of Molecular Genetics, Taussig Cancer Center, Lerner Research Institute, Cleveland Clinic Foundation, Cleveland, OH 44195, USA

**Keywords:** Rhabdomyosarcoma, NFκB, IKKβ, Cancer

## Abstract

Alveolar rhabdomyosarcoma (aRMS) is a pediatric soft tissue cancer commonly associated with a chromosomal translocation that leads to the expression of a Pax3:Foxo1 or Pax7:Foxo1 fusion protein, the developmental underpinnings of which may give clues to its therapeutic approaches. In aRMS, the NFκB–YY1–miR-29 regulatory circuit is dysregulated, resulting in repression of *miR-29* and loss of the associated tumor suppressor activity. To further elucidate the role of NFκB in aRMS, we first tested 55 unique sarcoma cell lines and primary cell cultures in a large-scale chemical screen targeting diverse molecular pathways. We found that pharmacological inhibition of NFκB activity resulted in decreased cell proliferation of many of the aRMS tumor cultures. Surprisingly, mice that were orthotopically allografted with aRMS tumor cells exhibited no difference in tumor growth when administered an NFκB inhibitor, compared to control. Furthermore, inhibition of NFκB by genetically ablating its activating kinase inhibitor, *IKKβ*, by conditional deletion in a mouse model harboring the *Pax3:Foxo1* chimeric oncogene failed to abrogate spontaneous tumor growth. Genetically engineered mice with conditionally deleted *IKKβ* exhibited a paradoxical decrease in tumor latency compared with those with active NFκB. However, using a synthetic-lethal approach, primary cell cultures derived from tumors with inactivated NFκB showed sensitivity to the BCL-2 inhibitor navitoclax. When used in combination with an NFκB inhibitor, navitoclax was synergistic in decreasing the growth of both human and *IKKβ* wild-type mouse aRMS cells, indicating that inactivation of NFκB alone may not be sufficient for reducing tumor growth, but, when combined with another targeted therapeutic, may be clinically beneficial.

## Abstract (precis)

**Summary:** In a genetically engineered mouse model of aRMS, disrupting the NFκB pathway facilitated tumor initiation, suggesting it is a modifier of the disease rather than the driver.

## INTRODUCTION

Rhabdomyosarcoma (RMS) is an aggressive soft tissue cancer affecting approximately 350 people in the United States annually (Breitfeld and Meyer, 2005; Reis LAG et al., 1999). RMS is one of the most common pediatric sarcomas and, when diagnosed at advanced stages, carries a dismal outcome. The disease is divided into two major subtypes, embryonal (eRMS) and alveolar (aRMS), the latter of which is believed to often arise from the *Myf6* myogenic lineage (Abraham et al., 2014; Keller et al., 2004a; Keller and Capecchi, 2004). aRMS is more aggressive than eRMS and displays a poorly differentiated phenotype (Qualman et al., 2008). aRMS tumors exhibit a unique genetic profile, with 85% of aRMS cases associated with a t(2;13) or t(1;13) chromosomal translocation that results in the fusion of the Pax3 or Pax7 DNA-binding homeo- and paired-domains to the transactivation domain of the transcription factor Foxo1 (Davis et al., 1994; Galili et al., 1993). This fusion protein drives tumor cells into a state recapitulating fetal myogenic precursors (Keller et al., 2004a,b; Keller and Capecchi, 2004). Despite intensive chemotherapy and radiation, aRMS carries only a 71% survival rate when localized. When metastatic, 5-year survival is below 20% (Ognjanovic et al., 2009). Despite scientific advances over the last 30 years, mortality and morbidity rates for RMS have remained stagnant (Malempati and Hawkins, 2012; Ognjanovic et al., 2009) and thus novel treatments are needed.

The NFκB transcription factor family is a highly conserved group of proteins consisting of RelA/p65 (p65), c-Rel, Rel-B, p60/p105 and p52/p100. These factors are maintained as homodimers and heterodimers, and are involved in a wide range of normal cellular processes such as differentiation, apoptosis, senescence, cell survival and immune responses, as well as aberrant cellular events sometimes leading to muscle disorders and oncogenesis (Bakkar et al., 2008; Di Marco et al., 2005; Dolcet et al., 2005; Guttridge et al., 1999; Mourkioti et al., 2006; Wang et al., 2009). These proteins all contain the same N-terminal Rel homology domain (RHD) necessary for DNA binding, dimerization, and interaction with inhibitory IκB proteins (Bakkar et al., 2008; Baldwin, 1996; Ghosh et al., 1998); however, only p65, c-Rel and RelB contain the transactivation domain required for transcription (Hayden and Ghosh, 2004). In an inactive state, NFκB dimers are bound to IκB factors such as IkBα or IkBβ, which mask the nuclear localization signal of the RHD, effectively retaining the inactive NFκB complex in the cytoplasm (Ghosh et al., 1998; Liou, 2002). The dimers are released and become transcriptionally active when the IκB protein degrades owing to phosphorylation by one of the IκB kinase (IKK) complexes, either IKKα, IKKβ or IKKϒ (Perkins, 2006).

Although NFκB is present and active in many different cell types, its role is particularly complex in skeletal muscle development and maintenance, where NFκB serves dual, complex functions as a result of two distinct activation pathways (Bakkar and Guttridge, 2010; Bakkar et al., 2008; Wang et al., 2009). The alternative NFκB signaling pathway regulates mitochondrial biogenesis, energy production and muscle homeostasis (Bakkar et al., 2008), and is mediated through IKKα phosphorylation of p100 (Xiao et al., 2004). The classical signaling pathway is activated by TNFα, followed by IKKβ phosphorylation of IκBα, resulting in translocation of p65 to the nucleus. This pathway acts during myoblast proliferation and utilizes several different mechanisms to prevent premature differentiation, either by transcriptional activation of cyclin D1 to maintain myoblasts in a cycling state (Dahlman et al., 2009; Guttridge et al., 1999), p65-mediated repression of *MyoD* mRNA or p65-mediated repression of muscle *miR-29* through the myofibrillar transcriptional repressor YinYang1 (YY1) (Wang et al., 2007). During normal myogenesis decreases in phosphorylated IκBα and phosphorylated p65 are observed, indicating a reduction in classical signaling, concomitant with increased alternative signaling as myoblasts begin differentiation and require energy from mitochondrial biogenesis (Bakkar et al., 2008; Guttridge et al., 1999). Increased levels of the p65 subunit of the NFκB complex have been reported in RMS cell lines and patient samples (Wang et al., 2008), suggesting that abnormal signaling of the transcription factor may play an oncogenic role in the disease.

Additionally, NFκB transcriptionally regulates Polycomb group member YY1 through binding of the p50/p65 subunit to the *YY1* promoter (Wang et al., 2007). YY1 epigenetically silences *miR-29*, which is decreased in RMS patient samples and cell lines, and retroviral delivery of *miR-29* to mice injected with Rh30 cells has been shown to slow tumor growth (Wang et al., 2008). These studies further implicate NFκB dysregulation in RMS.

Furthermore, high levels of the p65 subunit in RMS suggest activation of the classical pathway at a time during muscle development when the alternative pathway should be signaling. Although many cancers associate with classical signaling, the use of the alternative pathway in oncogenesis has been previously demonstrated (Demchenko et al., 2010; Nishina et al., 2009; Wharry et al., 2009). If the classical pathway is indeed active in aRMS, this offers a possible explanation for the characteristic undifferentiated morphology observed in patient tumors, the understanding of which may lead to novel therapeutic treatments of this disease.

Here, we attempt to elucidate the relevance of NFκB signaling in RMS initiation and progression, first by using pharmacological inhibition of the transcription factor *in vitro*. We additionally utilized a genetically engineered mouse model (GEMM) to understand the significance of genetic ablation of NFκB activity on tumor activation and progression, and analyzed combination therapy to potentiate NFκB inactivation.

## RESULTS

### Pharmacological NFκB inhibition reduces cell growth in a spectrum of soft tissue sarcomas

To investigate the role of NFκB in sarcoma, we first conducted a targeted chemical screen on 28 biologically independent mouse, canine and human cell lines and primary cell cultures, including aRMS, eRMS, undifferentiated pleomorphic sarcoma (UPS) and epithelioid sarcoma (EPS) (Table 1). We utilized the NFκB-inhibitor compound BAY 11-7082, which selectively inhibits TNFα phosphorylation of IκBα, effectively blocking the classical signaling pathway and preventing p65 translocation to the nucleus, resulting in abrogation of NFκB DNA-binding ability (Goffi et al., 2005; Lee et al., 2012). BAY 11-7082 resulted in a reduction of cell number in a variety of sarcomas when compared to untreated controls, as measured by CellTiter-Glo luminescent assay. This decrease in cell growth (IC_50_) was observed in most cases at concentrations that were comparable to NFκB-responsive cells in prior studies (8-10 µM) (Lee et al., 2012).

**Table 1.**
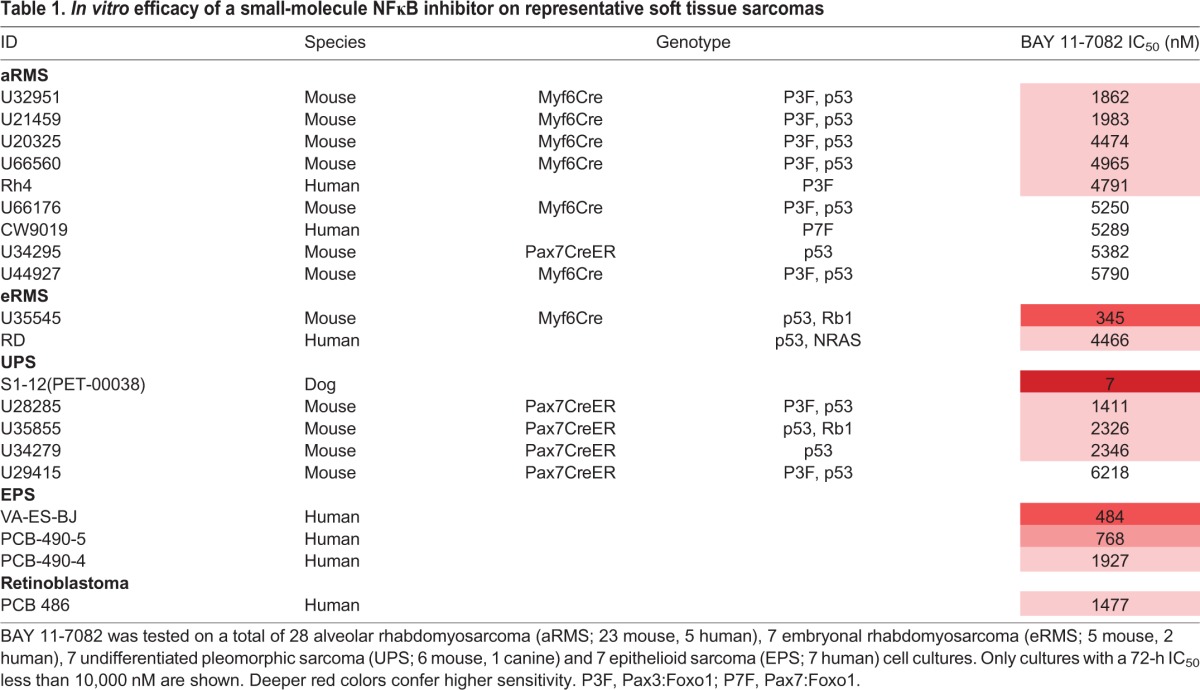
***In vitro* efficacy of a small-molecule NFκB inhibitor on representative soft tissue sarcomas**

### Pharmacological inhibition *in vivo* of NFκB is not efficacious in reducing tumor growth of alveolar RMS orthotopic allografts

Encouraged by the effect of NFκB inhibition on sarcoma tumor samples *in vitro*, we tested the efficacy of NFκB pharmacological inhibition *in vivo* using the NFκB essential modulator (NEMO)-binding domain (NBD) peptide, an independent and selective NFκB inhibitor. The NBD peptide is designed from the C-terminus of the IKKβ subunit and blocks activation of the IKK complex by interfering with IKK assembly (di Meglio et al., 2005; Strickland and Ghosh, 2006), thus diminishing NFκB transcriptional ability. NBD has previously been shown to be successful in blocking NFκB activation in mice (Reay et al., 2011).

**Fig. 1.**
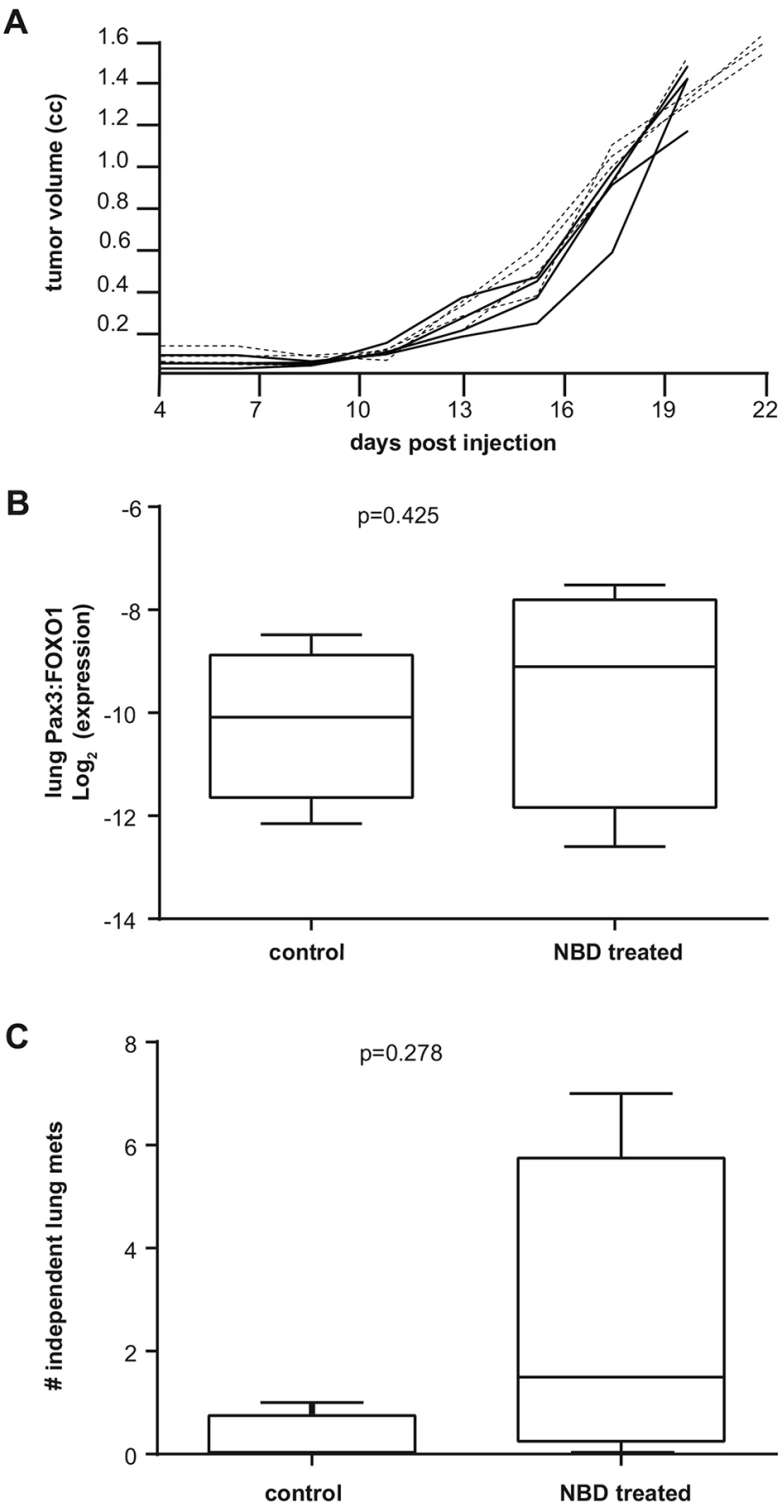
***In vivo* efficacy of an NBD peptide on aRMS.** (A) Tumor growth over time of SHO mice orthotopically allografted with aRMS tumor cells. Mice were treated with NBD peptide (black line; *n*=4; 10 mg/kg 3× per week by intraperitoneal injection) or vehicle (dashed line; *n*=4; 100 µl PBS 3× per week by intraperitoneal route), with endpoint measurement of tumor volume being 1.4cc. (B) qRT-PCR showing mRNA levels of *Pax3:Foxo1* in lungs of mice treated with vehicle or NBD peptide. (C) Number of independent lung metastases counted during histological analysis.

U48484 mouse aRMS cells harboring the *Pax3:Foxo1* chimeric oncogene were orthotopically allografted in the gastrocnemius muscle of SCID/hairless/outbred (SHO) mice and, when tumors reached 0.25 cm^3^, mice were treated with 10 mg/kg body weight NBD peptide, or vehicle, 3 times per week by intraperitoneal injection. Survival was not extended in the NBD-treated cohort, and tumor growth was not significantly reduced (Fig. 1A). To evaluate disease progression, reverse transcription PCR (RT-PCR) was conducted to examine the amount of *Pax3:Foxo1* mRNA in the lungs of NBD-treated and untreated mice. We found no significant difference in the total mRNA levels of *Pax3:Foxo1* in the lung, suggesting that the NBD peptide had no overt effect of reducing the rate of hematogenous metastasis (Fig. 1B). This result was confirmed by histological analysis, which showed no difference in the number of lung metastases between the groups (Fig. 1C).

### Genetic ablation of *IKKβ* causes highly aggressive RMS tumors with decreased latency

**Fig. 2.**
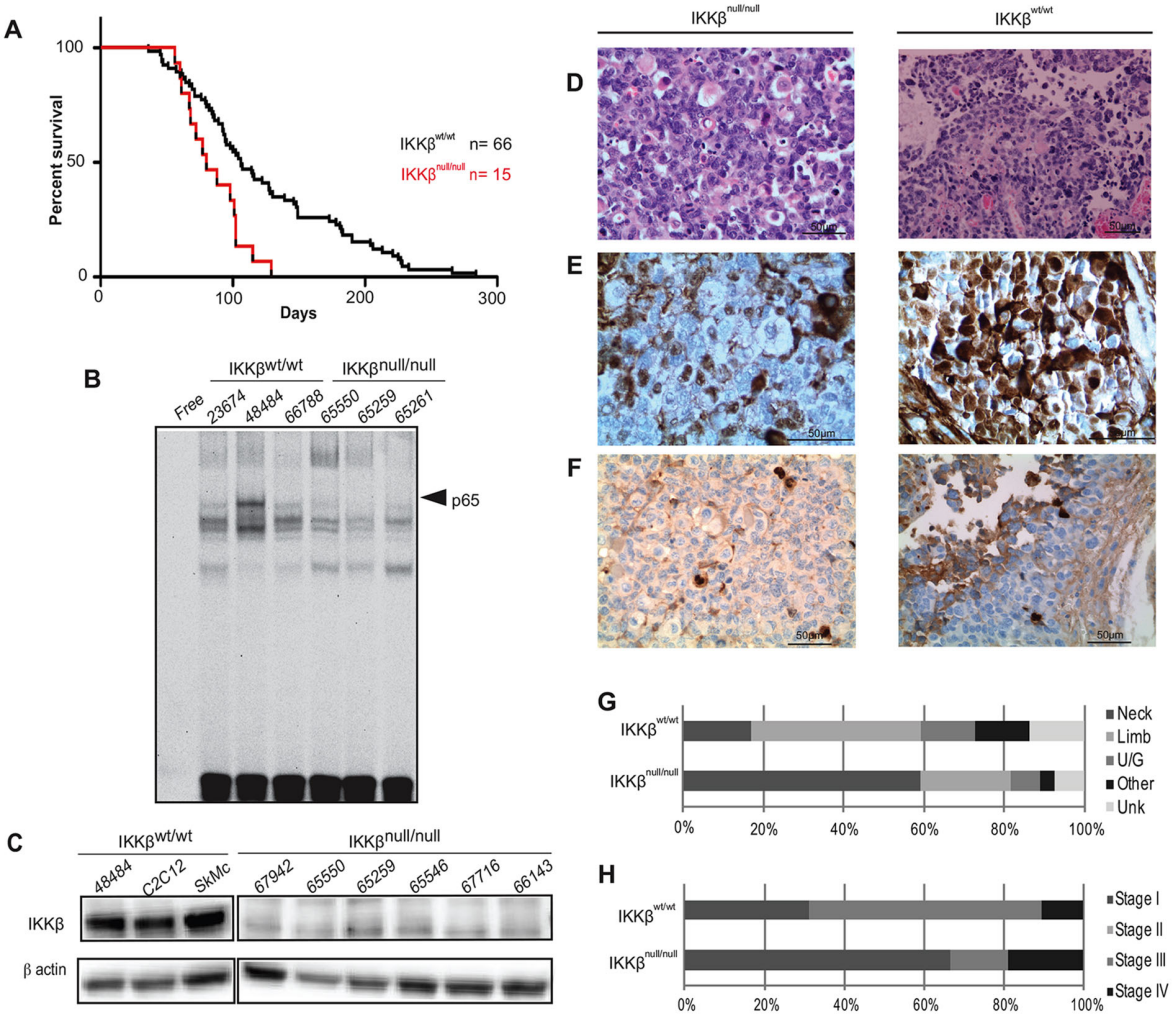
***IKKβ* deletion in aRMS.** (A) Kaplan–Meier survival curve of animals with *p53* inactivation and *Pax3:Foxo1* activation in the *Myf6Cre* lineage (*IKK*^wt/wt^) or *IKKβ* loss in combination with *p53* inactivation and *Pax3:Foxo1* activation (*IKK*^null/null^). The addition of *IKKβ* deletion to *Pax3:Foxo1*, *p53* mice significantly decreased tumor latency (paired *t*-test; *P*=0.0017). Conditional deletion of IKKβ protein was confirmed by western blot in all animals harboring the IKKβ^null^ allele. (B) EMSA performed with *IKK^wt/wt^* or *IKKβ^null/null^* cell extracts. Arrowheads denote p65/DNA bound complexes. (C) Representative western blot of IKKβ protein expression in aRMS mice with IKKβ^null^ allele. (D-F) Representative images of H&E (D), myogenin (E) or KI-67 (F) staining on tumors from *IKKβ*^null/null^ mice (U65261) compared with those from *IKK*^wt/wt^ control mice (U66564). (G,H) Anatomical site and tumor stage of tumors in *IKKβ*^wt/wt^ control mice compared to those with IKKβ deletion. U/G, urogenital; Unk, unknown.

To specifically test the role of NFκB on initiation and progression of the disease, we utilized a well-characterized aRMS mouse model that conditionally expresses the *Pax3:Foxo1* oncogene as a result of Cre recombination, and deletes *p53* (designated hereafter as IKKβ^wt^), in skeletal muscles. IKKβ^wt^ mice develop tumors 100% of the time and faithfully recapitulate the histological phenotype of the human disease. We crossed IKKβ^wt^ mice with mice that exhibit reduced NFκB activity owing to ablation of the IKKβ kinase in the presence of Cre recombinase (Acharyya et al., 2007; Mourkioti et al., 2006). The resulting mice harbor the Pax3:Foxo1 fusion protein, inactivated NFκB and deleted *p53* in skeletal muscles (designated hereafter as IKKβ^null^). Mice were viable and fertile, born in normal Mendelian ratios and developed normally through adolescence. When tumors developed, western blot analysis was performed on primary tumor cell cultures to confirm deletion of the IKKβ protein (Fig. 2C). Out of 17 primary cell cultures tested, 15 exhibited complete IKKβ ablation in the tumor, whereas 1 showed partial reduction, and 1 sample exhibited no decrease of IKKβ protein levels (Table S1). Some level of IKKβ protein from non-muscle cells was expected owing to the fact that protein was isolated from primary tumor cells that were cultured from whole tumor pieces, which harbor residual stromal and fibroblast cells.

Interestingly, IKKβ^null^ mice exhibited a significant decrease in tumor latency (log-rank test, *P*=0.0017, Fig. 2A) and a decrease in time from tumor onset to death compared to mice with active IKKβ. Fig. 2G,H show the anatomical site and surgical stage of IKKβ^null^ mice versus IKKβ^wt^. IKKβ^null^ mice exhibited a higher propensity for developing nonmetastatic stage-I tumors of the neck region than their wild-type counterparts.

To confirm that *IKKβ* deletion was having the intended effect on NFκB inactivation in genetically modified mice, nuclear extracts were prepared from IKKβ^wt^ and IKKβ^null^ primary tumor cell cultures and EMSA super-shift assay was performed. Results demonstrated that the p65 complex shifted only among the IKKβ^wt^ samples (Fig. 2B), indicating that deletion of IKKβ effectively prevents IKK-complex assembly, resulting in NFκB being relegated to the cytoplasm and unable to mediate transcription of target genes.

### IKKβ^null^ mice exhibit a histological phenotype similar to IKKβ^wt^

Hematoxylin and eosin (H&E) staining of IKKβ^null^ tumors showed a histological appearance similar to IKKβ^wt^ tumors (Fig. 2D). Tumors from IKKβ^null^ mice exhibited areas of rhabdomyoblastic differentiation mixed with cells generally negative for myogenin, whereas IKKβ^wt^ tumors consisted of characteristic clusters of small, round, myogenin-positive aRMS cells (Fig. 2E). Despite the aggressive nature of IKKβ^null^ tumors, Ki-67 staining showed no difference in proliferation index when compared to IKKβ^wt^ tumors (Fig. 2F), and no difference in frequency of rhabdomyoblasts between cohorts.

### IKKβ^wt^ primary tumor samples and cell lines are sensitive to combination therapy with a BCL-2 and NFκB inhibitor

**Fig. 3.**
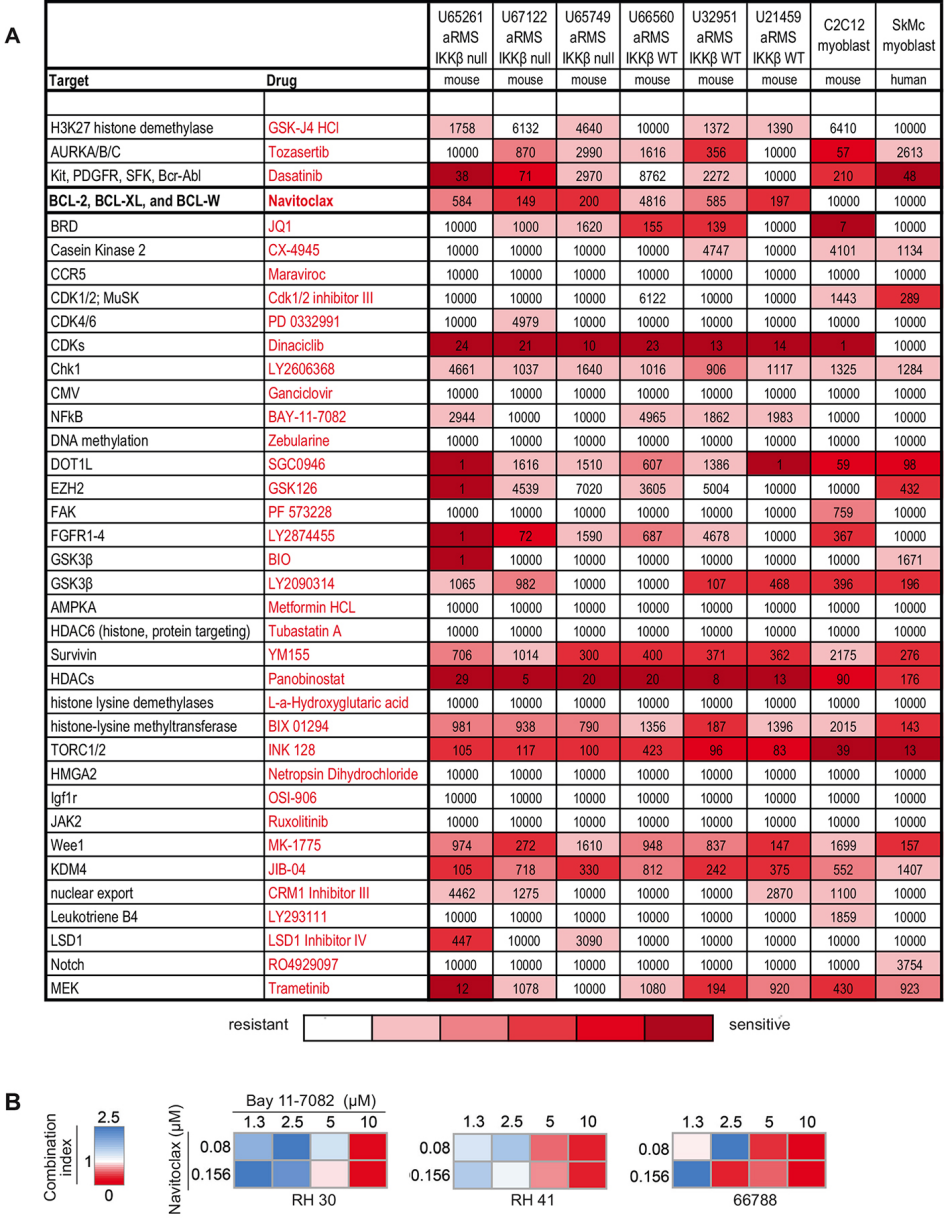
**Chemical screens for complementation of IKK genetic deletion.** (A) Navitoclax (bold) was efficacious in abrogating tumor cell growth in aRMS tumors that expressed both active and inactive NFκB. Deeper red colors confer higher sensitivity. All concentrations listed are in nM. The complete results are listed in Table S2. (B) Combination indices reflecting mouse and human aRMS cell lines. A combination index 0<1 represents a synergistic combination, 1 represents a neutral combination, and 1>2.5 is antagonistic. Synergistic combinations were achieved at doses within the reported active NFκB inhibitory range.

To investigate whether combination drug therapy could potentiate NFκB inactivation, we conducted a synthetic-lethal chemical screen intended to reveal novel targets in aRMS tumors with deleted *IKKβ*. Five of 6 tested aRMS tumor cell samples exhibited sensitivity to the BCL-2 inhibitor navitoclax (Fig. 3A), an orally bioavailable small-molecule protein inhibitor that is currently in Phase 1 trials for recurrent non-small-cell lung carcinoma and recurrent hepatocellular carcinoma, and Phase 2 clinical trials for platinum resistant/refractory ovarian cancer. The IC_50_ values for the 5 sensitive tumor cell samples treated with navitoclax ranged from 149 to 584 nM. We tested navitoclax in combination with the NFκB inhibitor BAY 11-7082 in the human aRMS cell lines Rh41 and Rh30, and mouse IKKβ^wt^ aRMS culture U66788 (Fig. 3B). Synergy (combination index <1) was detected in each of the three samples for navitoclax in the range of 0.08-0.156 µM and BAY 11-7082 in the range of 5-10 µM.

## DISCUSSION

We sought to explore the role of NFκB in aRMS disease progression, at first with a small-molecule compound, then with a peptide therapeutic, and finally by a genetic approach. The studies converged on the finding that canonical NFκB signaling plays no appreciable single pathway role in tumor progression. Interestingly, deletion of *IKKβ*, thereby inactivating classical NFκB signaling, facilitated tumor initiation, best characterizing its role as a cooperative initiating mutation/event.

The *Myf6Cre*, conditional *Pax3:Foxo1*, conditional *p53* (aRMS) GEMM is well characterized and has effectively demonstrated the requirement (or lack thereof) of various pathways for RMS progression. Notably, the addition of conditional *Rb1* loss to the aRMS GEMM was found to be a disease modifier but not sufficient for initiation of sarcomagenesis (Kikuchi et al., 2013), even though *Rb1* gene mutation is frequently reported in eRMS (Rubin et al., 2011; Kohashi et al., 2008). Additionally, targets such as PDGFRA, which are typically overexpressed in clinical cases of RMS, appear crucial to tumor progression when tested *in vitro* (Taniguchi et al., 2008). However, when conditionally deleted from the aRMS GEMM, *PDGFRA*-null mice exhibit an earlier onset and increase in tumor progression compared to those with intact *PDGFRA* (Abraham et al., 2012). *IKKβ* deletion was similarly informative in these studies.

Previous studies (Wang et al., 2008) were performed in Rh30, a 1987 cell line with gain of Pax3:Foxo1 and loss of p53 function, akin to our transgenic model. In those studies, miR-29b was overexpressed in the xenograft model and tumor growth slowed over a timescale of 20 days. The difference between these micro-RNA studies and the genetically deleted *IKKβ* studies might be explained by effects of miR-29b beyond NFκB signaling.

Our results point to NFκB still having a role in progression, but as a modifier of disease given that a synthetic-lethal interaction for Bcl2 inhibitors was seen for IKKβ^null^ aRMS primary cell cultures. This remains an area of open investigation.

## MATERIALS AND METHODS

### Drug sensitivity assays

For the BAY 11-7082 drug screen, mouse, human and canine primary tumor cell cultures were plated in a 96-well plate at 2500 cells/well. After 24 h, cells were incubated with varying concentrations of BAY 11-7082 (Selleckchem, Houston, TX, USA) for 72 h. CellTiter96 Aqueous One Cell Proliferation Assay (MTS) (Promega, Madison, WI, USA) was performed according to manufacturer's instructions, and a BioTek Synergy 2 plate reader (BioTek, Winooski, VT, USA) was used to evaluate the cytotoxic effect of the drugs. All concentrations were plated in triplicate. For synergistic combinational drug screens, mouse RMS primary tumor cell cultures (U65845; U66788) and human RMS cell lines (Rh30; Rh41) were plated and assayed as described above, with varying concentrations of Navitoclax (Selleckchem, Houston, TX, USA) or BAY 11-7082 (Selleckchem, Boston, MA, USA).

### Western blotting

Whole-cell lysate was taken from tumor cell cultures and cell lines when cells were 70% confluent at passage ≤5. Cells were rinsed with cold HyClone phosphate-buffered saline (PBS; Fisher Scientific, Waltham, MA, USA), scraped, and lysed with radioimmunoprecipitation (RIPA) buffer supplemented with a cocktail of protease inhibitors and serine/threonine and tyrosine phosphatase inhibitors (Fisher Scientific). Protein supernatants were separated by 7.5% Mini-Protean pre-cast gels (Bio-Rad, Hercules, CA, USA) at 120 V for 1.5 h then transferred onto a PVDF membrane at 100 V for 1 h. The membrane was then blocked with 5% nonfat skim milk in TBS-T for 1 h then incubated overnight in primary antibody. Primary antibodies used were: mouse β-actin (1:10,000, A1978, Sigma Aldrich, St Louis, MO, USA); mouse IKKβ (1:250, IMG-129A, Novus Biologicals, Littleton, CO, USA); mouse myosin heavy chain (clone MF20) (1:500, MAb4470, R&D Systems, Minneapolis, MN, USA); and phospho-p65 (1:1000, 4025, Cell Signaling, Danvers, MA, USA).

### Cell culture

All mouse derived primary tumor cell cultures were generated as previously described (Taniguchi et al., 2008) and used at passage <5. Briefly, tumors were digested with 1% collagenase IV (17104019, Sigma Aldrich, St Louis, MO, USA) in Gibco Dulbecco's modified Eagle's medium (DMEM) (11965092, Thermo Fisher Scientific, Waltham, MA, USA) overnight then passed through a 70 µM cell strainer into a 10 cm tissue culture treated dish. Cells were maintained in DMEM supplemented with 10% fetal bovine serum (FBS; 10438034, Thermo Fisher Scientific) and 1% Gibco PenStrep (15140122, Thermo Fisher Scientific) at 5% CO_2_ in air at 37°C. The human RMS cell lines Rh30 and Rh41 were generously provided by the Houghton Laboratory at St Jude's Cancer Research Hospital (Memphis, TN, USA). Human SkMc cells were obtained from Lonza (cc-2561, Walkersville, MD, USA). C2C12 mouse myoblast cells were purchased from the American Type Culture Collection (CRL-1772, Manassas, VA, USA).

### Mice

All animal procedures were conducted in accordance with the Guidelines for the Care and Use of Laboratory Animals and were approved by the Institutional Animal Care and Use Committee (IACUC) and housed at Oregon Health & Science University. The *Myf6Cre*, conditional *Pax3:Foxo1*, conditional *p53*, and IKKβ^null^ mouse lines and corresponding genotyping protocols have been described previously (Keller et al., 2004a; Keller and Capecchi, 2004; Mourkioti et al., 2006; Nishijo et al., 2009; Pasparakis et al., 2002). Owing to the sudden onset and aggressive nature of these tumors, tumor-prone mice were visually inspected every 2 days. Tumor staging was based on a previously described adaptation of the Intergroup Rhabdomyosarcoma Study Group Staging system.

### *In vivo* study with NBD peptide

Female SCID/Hairless/Outbred mice were purchased from Charles River Laboratory (Crl:SHO*-*Prkdc^scid^ Hr^hr^, Wilmington, MA, USA) at 8 weeks of age and were injected with cardiotoxin (217503, EMD Millipore, Bellerica, MA, USA) into the gastrocnemius muscle. After 24 h, 1×10^6^ U48484 mouse aRMS cells were injected into the same muscle. Tumor volume measurements were taken 3 times weekly and, when tumor volume reached 0.25 cm^3^, mice were given either 10 mg/kg body weight NBD peptide or an equal volume of PBS vehicle by intraperitoneal injection every other day. When tumor volume reached 1.5 cm^3^, mice were humanely euthanized and tissue samples were collected.

### RNA isolation and quantitative RT-PCR (qRT-PCR)

Probes set for mouse tissue samples were *Gapdh*-Mm99999915_g1, and *Pax3:Foxo1* 5′-6-FAM-AATTCGCCACCAATCTGTCCCTTCA-TAMRA-3′. From whole-tumor chunks, total RNA was isolated using Trizol (15596018, Thermo Fisher Scientific) following the manufacturer's instructions. The RNeasy mini kit (74104, Qiagen, Valencia, CA, USA) was then used to process RNA to cDNA. Mouse *Gapdh* was used as a control for relative gene expression and the mean of three experimental replicates per specimen was used to calculate the ratio of gene of interest/*Gapdh* expression for the Taqman assay using Bio-Rad CFX Manager software.

### EMSA super-shift assay

EMSA was performed as previously described (Guttridge et al., 1999). Briefly, nuclear extracts were prepared from *IKKβ*^wt/wt^ and *IKKβ*^null/null^ mice and incubated with 20,000 cpm of radiolabeled probes. A rabbit polyclonal antibody against the p65 subunit (100-4165, Rockland, Gilbertsville, PA, USA) of NFκB was incubated with nuclear extracts for 15 min prior to the addition of poly(dI-dC) and a ^32^P-labeled probe. Complexes were resolved on a 5% polyacrylamide gel in Tris-glycine buffer (25 mM Tris, 190 mM glycine, 1 mM EDTA) at 25 mA for 2-3 h at room temperature. The gels were dried and exposed on film for approximately 1-3 days.
